# Lesion-network mapping in task-dependent frequencies uncovers remote consequences of focal damage

**DOI:** 10.1162/imag_a_00557

**Published:** 2025-04-30

**Authors:** Alireza Chamanzar, Erez Freud, Pulkit Grover, Marlene Behrmann

**Affiliations:** Electrical and Computer Engineering Department, Carnegie Mellon University, Pittsburgh, PA, United States; Neuroscience Institute, Carnegie Mellon University, Pittsburgh, PA, United States; Department of Pathology, Computational Pathology & AI Center of Excellence (CPACE), University of Pittsburgh School of Medicine, Pittsburgh, PA, United States; Department of Psychology and the Centre for Vision Research, York University, Toronto, Canada; Department of Psychology, Carnegie Mellon University, Pittsburgh, PA, United States; Department of Ophthalmology, University of Pittsburgh School of Medicine, Pittsburgh, PA, United States

**Keywords:** visual agnosia, EEG, object recognition, ventral/dorsal pathways, diaschisis, neuropsychology

## Abstract

The brain consists of a multiplicity of networks with massively interacting nodes. Disruption of a node following brain damage can result in both short- and long-distance functional abnormalities, affecting even intact brain regions remote from the site of lesion (termed ‘diaschisis’). Diaschisis has been well described previously, and structural and functional connectivity have been related to clinical findings. However, the mechanistic and neurophysiological properties of this remote loss of function, its temporal and spectral dynamics, and its impact on the whole brain remain to be elucidated. In this study, we used high-density electroencephalography (EEG) to detect and characterize function- and frequency-dependent transcallosal diaschisis in a single-case of visual agnosia who has a perceptual deficit in object and face recognition following a focal lesion in the right posterior temporal cortex. Scalp EEG activity was evoked by images of intact and parametrically increased scrambled objects. SilenceMap, an algorithm developed for the location of reduced power (i.e., regions of silence), was used to estimate the slope of shape-selective EEG responses at levels of object scrambling, with structural and functional MRI serving as the ground truth for the lesion and diaschisis. The functional deficit, manifest as a significant reduction in the slope of EEG object shape sensitivity, was observed in the lesioned right ventral cortex and right dorsal cortex across most of the frequency bands (>4  Hz). This reduction in EEG slope was accompanied by contralesional diaschisis in the homotopic left ventral and left dorsal cortex but only in the Theta band (4​−​8Hz). This noninvasive approach both elucidates the neural correlates of diaschisis and confirms the viability of this approach in identifying neurological abnormality, perhaps offering a path toward precision medicine.

## 1 Introduction

In recent years, there has been growing recognition that the brain does not comprise independent, discrete components but, rather, functions as a dynamic and distributed system with massively interacting neurons and networks (Barabási et al., 2023). This large-scale network organization is considered beneficial for neural computation, with disparate regions contributing different constraints and representations. While the benefits of a distributed system might be obvious, there is also a cost—namely, following damage to one node, there is a ripple effect on remote nodes, impairing function even at distal sites (Siegel et al., 2022). This remote effect was termed ‘diaschisis’ (from the Greek ‘dia’ meaning ‘in half’ or ‘across’ and ‘schizien’ meaning ‘to split’) by von Monakow (Finger et al., 2004). Diaschisis is assumed to occur via afferent terminals from the lesioned area, in concert with functional and/or metabolic changes (Salvalaggio et al., 2020), all of which adversely impact neurobehavioral function (Nguyen & Botez, 1998).

### 1.1 Cortical diaschisis in humans

The remote effect may result from acute or chronic degenerative etiologies and may implicate cerebellar and/or thalamic connectivity with cortex (Nguyen & Botez, 1998). Subcortical-to-cortical forms of diaschisis have received attention in recent years (Sommer et al., 2016;Zhang et al., 2023) and, over many years, studies of cortico-cortical transcallosal forms of diaschisis (Carrera & Tononi, 2014) have revealed bihemispheric reduction of blood flow and metabolism in, for example, motor (Hensel et al., 2021) and visual (Rizzo & Robin, 1996) activity (for review, seeCampos et al., 2023;Feeney & Baron, 1986). In these latter studies, interhemispheric diaschisis is usually documented using positron emission tomography (PET) scanning or functional magnetic resonance imaging (fMRI) as measured during resting state (Fiorelli et al., 1991) or task-evoked activities (Corbetta et al., 2018). These imaging modalities (Carrera & Tononi, 2014), together with multivariate analyses and graph theoretical characterization of the neural activity (Siegel et al., 2022) have uncovered brain-wide connectivity disruptions (Corbetta et al., 2018) and opened new pathways for the examination of diaschisis.

One provocative property of diaschisis is that it may explain how a unilateral lesion mimics the behavioral profile observed after a frank bilateral lesion (De Renzi, 2000;De Renzi et al., 1991). For example, in a patient with “visual agnosia” (SM), the visual recognition deficit was so striking that, at first, bilateral damage seemed likely. On imaging, however, the patient had a small (990 mm^3^), circumscribed unilateral lesion to the right lateral fusiform gyrus in the vicinity of the Lateral Object Complex (LOC) (Konen et al., 2011). Altered neural function was, however, also apparent in the homologous region of the preserved hemisphere. Interestingly, this diaschisis was task-dependent (Konen et al., 2011), in that it was apparent to a greater degree as task complexity increased: in a univariate fMRI contrast of viewed objects versus fixation, reduced BOLD signal was observed in the lesion site but no diaschisis was evident in the homotopic left LOC. When SM was required to discriminate between objects, however, or was shown repeated versus non-repeated trials to elicit adaptation or ‘repetition suppression’, activation in the homotopic left LOC region was markedly atypical relative to controls. Further investigation of the SM’s entire visual cortex also uncovered abnormally low activation in the dorsal visual cortex in both the lesioned right hemisphere (RH) and in the structurally preserved left hemisphere (LH) in response to displayed objects (Freud & Behrmann, 2020). Given the tight coupling of ventral and dorsal visual pathways (Yeatman et al., 2014), the unilateral RH ventral lesion likely adversely affected the LH ventral region with knock-on diaschisis effects in both the preserved RH and LH dorsal regions. The unique profile of within- and between-hemisphere signal reduction in this individual offers a unique testbed to explore further the detailed characteristics of the diaschisis in the distributed object recognition network (Ayzenberg & Behrmann, 2022;Holler et al., 2019). This patient served as the participant of the current investigation.

That diaschisis is possible in regions remote from the cortical damage is incontrovertible. However, with some exceptions, there have been rather few attempts to offer a mechanistic account of the remote impact beyond one of inhibition from afferent terminals from the lesioned area (Rosenzopf et al., 2023). Quantifying the electrophysiological properties of diaschisis by elucidating the spectral and temporal dynamics by which the remote area is affected, and documenting the particular conditions or contexts under which diaschisis is manifest remain to be accomplished.

### 1.2 Quantifying the electrophysiological properties of diaschisis

One approach to quantifying the diaschisis is to examine frequency-specific effects of the signal from the lesioned area to the remote affected region. Whereas faster oscillations corresponding to local activity are especially evident in the domain of primary sensory processing, which is connected by thick highly myelinated fibers, lower-frequency oscillations correspond to more remote synchronization via thinner, less myelinated fibers connecting associated cortices (Aboitiz et al., 1992;Gong & Zuo, 2023). Specific profiles of cognitive functions can also be explored, including oscillations in the Beta frequency band, 15–30 Hz, which affect long-distance functional connections as well as working memory (Bassett et al., 2009), and oscillations at multiple frequencies (including Theta, Alpha, and low Beta) which are mostly visible in early visual areas and are correlated with perceptual tasks such as face recognition (Caplette et al., 2023;Gallina et al., 2024) (for a review of cortical circuits and oscillations, see, e.g.,Miller & Buschman, 2013). Most relevant here, is that, while Theta band activity is widespread in visuospatial tasks and in short-term memory tasks, during object recognition, Theta is more restricted (and elevated) in the inferior temporal cortex (Snipes et al., 2022). In sum, here, we measure the frequency band profile in the site of the patient’s lesion and predict that Theta band signal may reveal parameters of the inhibitory effect of the lesion and its cascade through the network of connected regions.

In the current study, using high-density EEG, we undertake a detailed characterization of cortico-cortical abnormalities of signal propagation within- and between-hemispheres. We compare whole-brain EEG data acquired during a parametrically manipulated object recognition task from SM, a patient with marked visual agnosia, as mentioned above, and a group of age- and gender-matched controls. This permits the investigation of possible functional or task differences in the diaschisis. Importantly, we compare the EEG findings with ground truth revealed by SM’s documented structural MRI lesion and compare it to the known diaschisis in fMRI activation evident in both dorsal and ventral cortex in both hemispheres (Freud & Behrmann, 2020;Konen et al., 2011). Specifically, here, we examine both frequency- and task-specific effects of this “dynamic diaschisis” (Price et al., 2001).

To preempt our conclusions, we document altered EEG signal in the lesioned right ventral cortex and in the right dorsal cortex. Signal perturbation was also uncovered in the left hemisphere ventral and dorsal areas, consistent with the fMRI ground truth (Freud & Behrmann, 2020;Konen et al., 2011). Compared with controls, the EEG signals in all four (dorsal/ventral x right/left) areas in SM evinced significantly less sensitivity to the stimulus manipulation (parametrically scrambling the object input). As a means of exploring the diaschisis further, we map out the spectral differences measured around the lesion, and in the more remote regions, as well, and while many frequency bands are implicated, alterations in Theta band are the most pronounced.

## 2 Methods

### 2.1 Participants

A single patient with a unilateral circumscribed ventral visual cortex lesion and five matched control participants were recruited. All participants had normal or corrected-to-normal vision by self-report and received payment for their participation. This protocol was approved by Carnegie Mellon University Institutional Review Board (IRB HS13-666 for the controls, and IRB HS14-607 for the patient “Visual recovery after severe brain injury or visual pathway disturbance”), and participants provided informed consent to participate.

#### 2.1.1 Case study

SM, a 47-year-old male, suffered a closed head injury in a motor vehicle accident at the age of 18. Clinical CT scans acquired close to the time of the injury indicated a contusion in the right anterior and posterior temporal cortex. A 3T MRI scan from 2009 revealed a circumscribed lesion in the right occipito-temporal cortex in the vicinity of area LOC (for detailed lesion demarcation, seeFig. 1a). SM is right-handed (but sometimes uses his left as a result of some damage to the right hand), has corrected-to-normal vision, and completed high school (Freud & Behrmann, 2020;Konen et al., 2011). He has been employed in the family business following the accident and after rehabilitation. SM’s visual abilities have been described in detail previously (Freud et al., 2017c;Gilaie-Dotan et al., 2013,^2015^;Marotta et al., 2001;Nishimura et al., 2010), and detailed data confirming that his prosopagnosia and object agnosia are available, as wellBehrmann and Kimchi (2003)andBehrmann et al. (2005). We confirmed the presence of visual agnosia in a behavioral test which was used in the previous study (seeFig. 2afor details, adopted fromFreud & Behrmann, 2020).

**Fig. 1.**
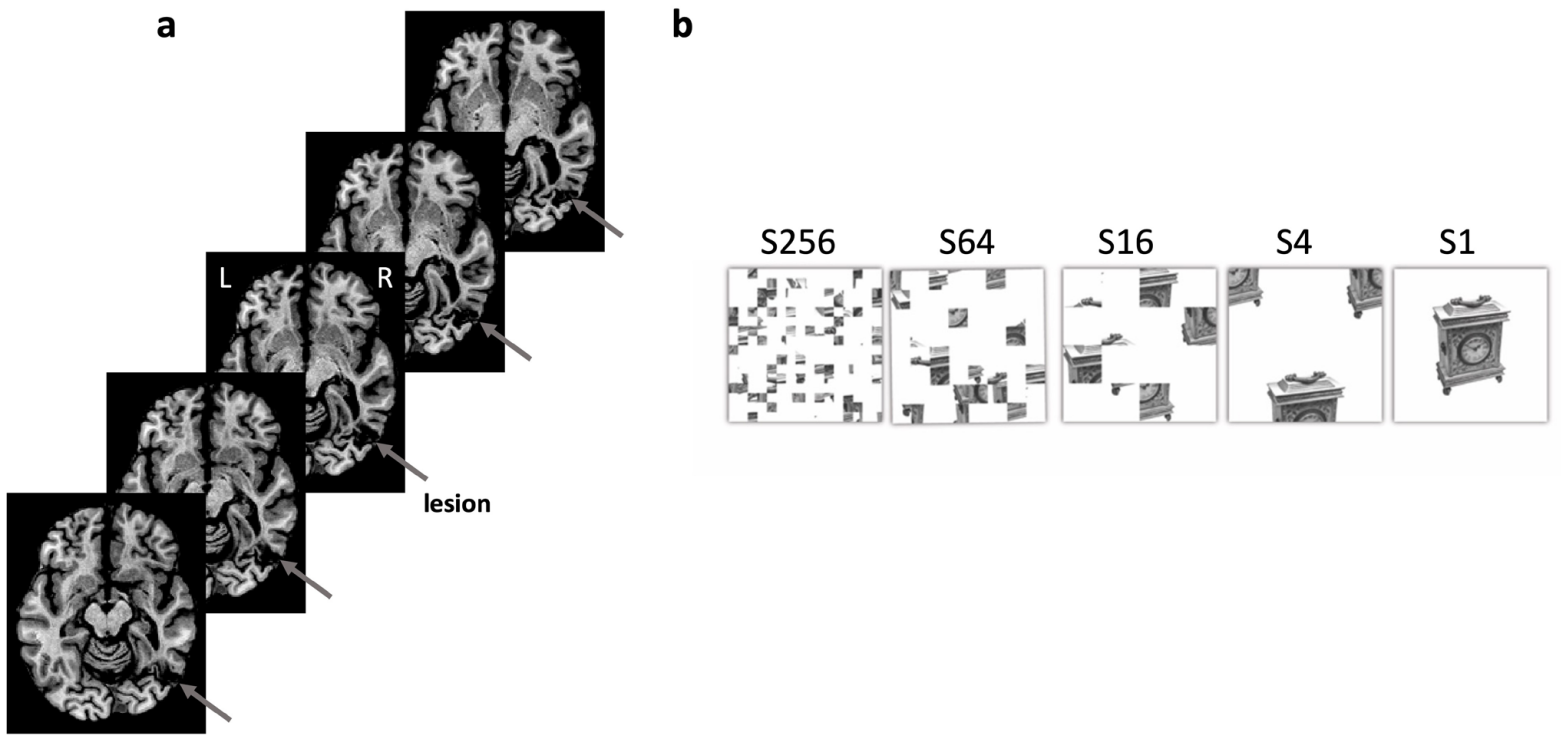
(a) Axial slices of structural T2 MRI scan with the gray arrow indicating the RH posterior ventral lesion. From lower left to upper right, slices are located at -11 to -7 MNI Z-coordinate. (b) The experimental stimuli consisted of images of intact real objects which were manipulated by dividing up the display using an invisible grid of different sizes (from 4 to 256 cells) and then randomly rearranging the squares.

**Fig. 2.**
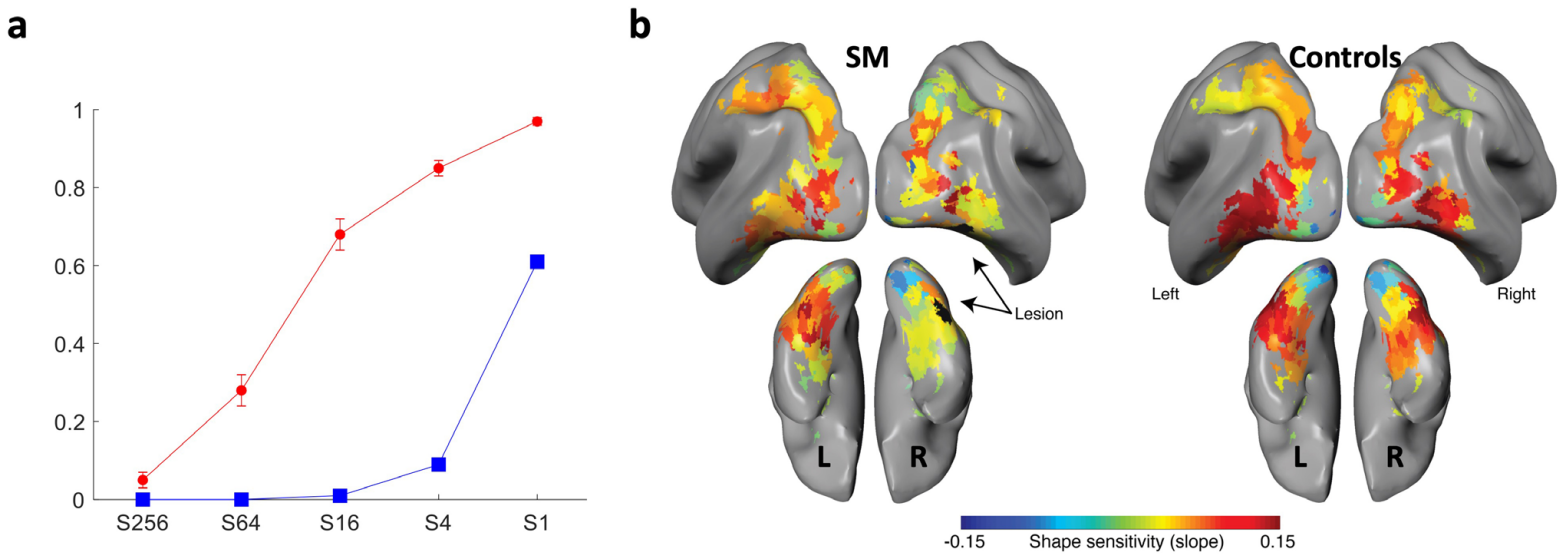
(a) Accuracy of object recognition (verbal response) for SM and controls. SM shows lower accuracy than the controls for almost all of the scrambling levels, except S256 which is at floor level for the controls. SM is also more affected by the scrambling manipulation, that is, the decrement in SM’s accuracy is sharper than the decrement in the controls. Adapted fromFreud and Behrmann (2020). (b) Slope in shape sensitivity (defined as slope across increasingly scrambled visually presented objects) in SM (on the left) versus a group of matched controls (on the right), measured using BOLD responses (Freud & Behrmann, 2020;Freud et al., 2017b). The lesion extent in SM is colored black and denoted by the arrows. Adapted fromFreud and Behrmann (2020). In comparison with controls, SM shows a clear reduction in the slope across scrambling level in the locus of the RH lesion and in the homologous location in the structurally intact LH. In addition, the posterior parietal lobes in the RH and LH also show evidence of shape sensitivity reduction, further indicative of diaschisis. These fMRI results are used as the ground truth for the EEG portion of this paper. The control data in this figure includes participants fromFreud and Behrmann (2020)andFreud et al., (2017b).

Importantly, SM has normal sensory vision and intact low-level perception (e.g., edge orientation and color perception). SM can also perform simple Gestalt grouping operations such as closure and collinearity, but is unable to apprehend a multi-element stimulus as a whole shape unless there is strong perceptual support for grouping or sufficient time for him to “solve the puzzle” (his words) (Behrmann & Kimchi, 2003).

A focal lesion with a volume of 990 mm^3^(Talairach coordinates +44, 46, 2 (Talairach, 1988)) was identified in the posterior part of SM’s RH lateral fusiform gyrus (Freud & Behrmann, 2020;Konen et al., 2011). Functionally, the lesion is located within LOC, which is typically defined in fMRI studies by contrasting BOLD responses to objects versus scrambled images. SM has had multiple functional and structural MRI scans (Freud & Behrmann, 2020;Freud et al., 2017c). Some of the MRI data were acquired at the CMU-Pitt BRIDGE Center (RRID:SCR_023356). Of specific relevance for this investigation, in one fMRI study, we mapped activation in the RH lesion and its penumbra and in the LH homotopic region with increasingly challenging visual input (intact objects which are then scrambled into smaller and smaller components, seeFig. 2b). The reduction in BOLD, within the RH lesion and in the homologous location in the structurally intact LH, was task-dependent and specific to these regions. In both hemispheres, retinotopic mapping obeyed typical topographic organization (Konen et al., 2011). When dorsal and ventral cortex was examined in a subsequent fMRI study, reduced BOLD signal was also evident in the RH posterior parietal lobe as well as in the homologous LH dorsal region (Freud & Behrmann, 2020).

#### 2.1.2 Control participants

Five age- and gender-matched individuals (mean age 45 years; range 41–50 years) were recruited to participate in this study. All participants were right-handed, and none had a history of neurological disorder.

### 2.2 Experimental protocol

Because we intended to compare the diaschisis in EEG that previously observed in BOLD, we replicated the experimental protocol used for SM’s BOLD study (Freud & Behrmann, 2020) using EEG. We present details here and the reader is referred to the previous paper (Freud & Behrmann, 2020) for additional information.

Briefly, the stimuli were 80 grayscale pictures of 40 everyday objects and 40 tools identical to those used previously (Freud & Behrmann, 2020;Freud et al., 2017b). To manipulate the integrity of the image, we employed a box-scrambling algorithm that divided each intact image into 4, 16, 64, and 256 squares and randomly rearranged the squares, resulting in five levels of scrambling, labeled S1 (intact), S4, S16, S64, and S256 (seeFig. 1b). In separate blocks, participants viewed displays at one of the five levels of scrambling (S256, S64, S16, S4, and S1 (intact)) blocked by category (tools or objects). Each picture was presented for 800 ms, with a variable interstimulus interval (ISI) between 600–1000 ms. The order of the blocks was consistent across participants. Object scrambling is a simple, but highly effective paradigm that is known to modulate perceptual abilities as well as the neural responses of regions in the dorsal and ventral pathways (Freud et al., 2017a;Lerner et al., 2001). We chose this task specifically to evaluate whether, using scalp EEG, we could converge on the same findings already reported using structural and functional MRI (Freud & Behrmann, 2020). This provides a proof of concept of the approach adopted here. In addition to this, by exploiting the high temporal resolution EEG data, we were able to obtain a systematic and deeper understanding of the spectral and temporal profile of the lesion and remote areas. In addition to the object scrambling task, we also recorded EEG during three blocks of 120 s resting state in which a fixation cross was presented in the center of the screen with a gray background.

In a separate behavioral task, conducted outside the scanner, recognition of these displays was measured by showing the stimulus on a computer screen for 600 ms, and requiring verbal naming of the object. SM’s performance was significantly different from that of the controls (Freud & Behrmann, 2020). SM’s accuracy decreased disproportionately as the scrambling level increased, with only 61% accuracy on the intact object condition compared with the control mean of 97%. SM’s recognition at the S4 condition reached only 9% whereas the controls’ mean was roughly 80% (seeFig. 2afor details, adopted fromFreud & Behrmann (2020)). These data verify SM’s visual agnosia on the very objects displayed during the EEG task used here.

### 2.3 EEG data acquisition

The data were acquired while the intact and scrambled objects were viewed and also during resting state on a BioSemi Active Two system (BioSemi, Amsterdam, Netherlands). Data were sampled at 512 Hz with a 24-bit A/D conversion. We used a 10-5 standard high-density electrode montage (Oostenveld & Praamstra, 2001) with 128 electrodes secured in place using a nylon head cap. Four electrodes were added around the eyes: one above and one below the right eye, and two at the outer canthi of each eye to monitor eye movements. An additional electrode was added to the left collar bone to detect heartbeat. All electrodes were recorded relative to the standard BioSemi CMS and DRL electrodes.

### 2.4 Data analysis

#### 2.4.1 Prepossessing steps

Preprocessing used EEGLAB (Delorme & Makeig, 2004) toolbox in MATLAB and followed the steps inChamanzar et al. (2021): We bandpass filtered the EEG signals in the range of[1,50]Hz. We then visually identified the noisy channels, removed them, and spatially interpolated them (8 channels in SM, up to a maximum of 9 channels in controls). Thereafter, we calculated differential channels for vertical and horizontal eye movements using the data from the electrodes placed around the eyes and, together with the heart channel and data from the 128 scalp electrodes, performed an independent component analysis (ICA) to extract and remove artifacts from the EEG recordings. We examined the quality of the channels one more time following the artifact removal, pruned the signal based on abnormal trends in the channel statistics (seeChamanzar et al., 2021for more details), and removed noisy time windows through visual inspection of the continuous recordings. We bandpass filtered the preprocessed data in standard EEG frequency bands of Delta ([1,4]Hz), Theta ([4,8]Hz), Alpha ([8,12]Hz), Beta ([12,30]Hz), and Gamma ([30,50]Hz) for spectral power analysis of differences between SM and controls.

#### 2.4.2 SilenceMap overview

**Fig. 3.**
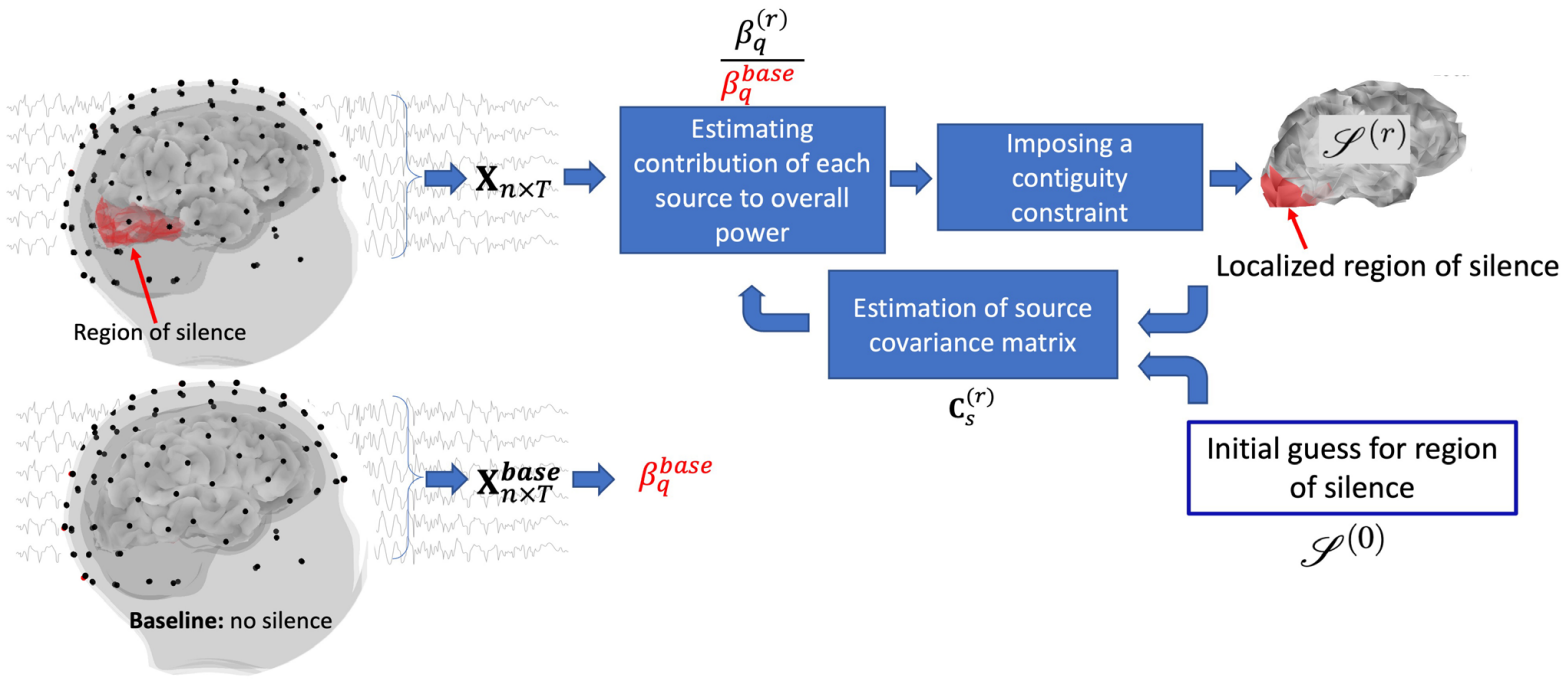
Overview of the main steps in SilenceMap (Chamanzar et al., 2021): this is an iterative approach for noninvasive localization of regions of silence in the brain ($S^{\left(r\right)}$, at iteration r) using scalp EEG recordings (${\text{X}}_{n\times T}$, where n is the number of EEG electrodes and T is the number of time points). SilenceMap measures the contribution of activity ($\beta_{q}^{\left(r\right)}$) at each source location in the brain to all EEG signals on the scalp in the power sense. This is done using the EEG recordings and an estimate of source covariance matrix (${\text{C}}_{s}^{\left(r\right)}$) with a contiguity constraint to localize a single region of silence in each hemisphere in the brain. A baseline recording (${\text{X}}_{n\times T}^{base}$) is used to normalize the source contribution measures and permit localization of the region of silence.

SilenceMap estimates the location of sources in the brain with reduced power (i.e., region of silence$S^{\left(r\right)}$) using scalp EEG (Chamanzar et al., 2021). The algorithm involves iterations (r) during which the contribution of activity at each source is localized in the brain to the entire EEG power on scalp. This is done using the scalp EEG signals (${\text{X}}_{n\times T}$, where n is the number of scalp electrodes and T is the number of time points) and an estimation of source covariance matrix (${\text{C}}_{s}^{\left(r\right)}$). The source covariance matrix alone does not reveal the location of silent sources as the problem is under-constrained and many solutions are possible. To constrain the solution, assumptions about the nature of the region of silence are made, for example, that the region of silence is a contiguous region. Finally, through iterations with this contiguity constraint, we keep updating the source covariance estimate and localize the region of silence, until it converges to a location in the brain. This process is done for each hemisphere separately to localize potential regions of silence in each hemisphere. SilenceMap needs a baseline (${\text{X}}_{n\times T}^{base}$) to reliably localize the region, and the estimated source contribution measures ($\beta_{q}$’s) are normalized using this baseline.Figure 3summarizes these main steps of SilenceMap.

This approach enabled us both to solve the inverse problem of signal from scalp to the silent region in the brain and to estimate a measure of activity using spectral power rather than just the average amplitude. We can estimate and localize power abnormalities in different frequency bands (i.e., oscillations) without requiring that the neural responses be phase-synchronized across trials.

*Estimation of source contribution measures (β).*For each of the frequency bands (Delta, Theta, Alpha, Beta, Gamma), at each level of scrambling (S256, S64, S16, S4, and intact), we estimated the contribution of all sources in the brain to the measurements on the scalp (Chamanzar et al., 2021). The derivedβcoefficients permit the calculation of shape sensitivity as the slope across the different levels of scrambling per frequency band.

**Fig. 4.**
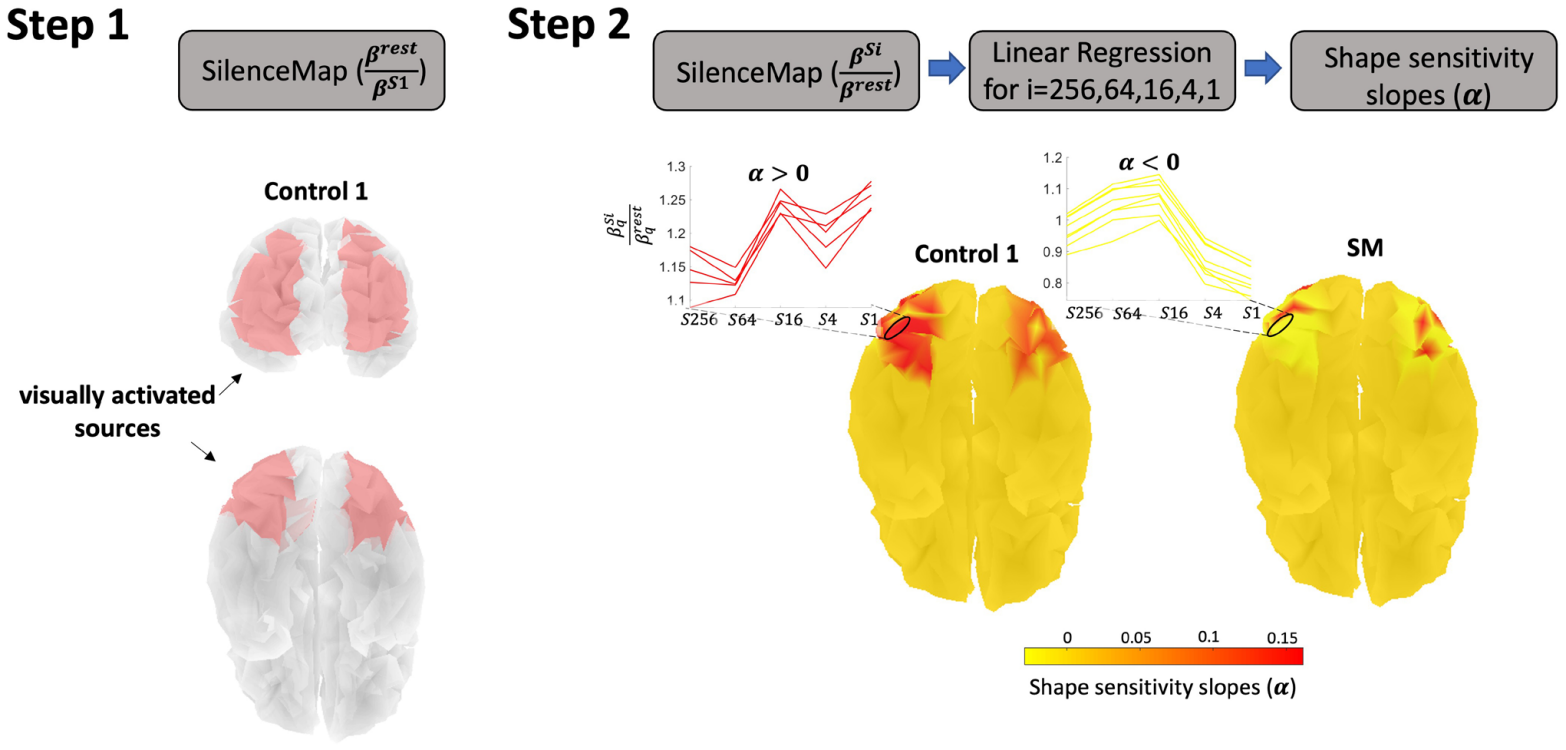
Main steps for shape sensitivity estimation using SilenceMap: Step 1. Localization of visually activated sources in a healthy control. We refer to these regions as Level 1 regions of interest or ROIs (seeTable 1for details). SilenceMap is used to localize contiguous regions with increased source contribution measure (i.e.,$\beta^{\text{S}1}$) using just the intact object condition, in comparison to the resting state (i.e.,$\beta^{\text{rest}}$). Note that SilenceMap can only localize a single contiguous region in each hemisphere. Step 2. For these visually responsive sources, shape sensitivity slopes (α) are estimated. To ensure statistical independence in definition/localization of visually activated regions and data extraction, the regions are localized in the healthy controls and then used for analysis of SM’s data. Using SilenceMap, source contribution measures of the sources for different object scrambling levels (S1, S4, S16, S64, S256) are estimated, normalized by the resting state (i.e.,$\frac{\beta_{q}^{Si}}{\beta_{q}^{rest}}$). Using a linear regression, the slope of$\frac{\beta_{q}^{Si}}{\beta_{q}^{rest}}$acrossi=256,64,16,4,1is estimated for each sourceqin the visually activated region. These estimated slopes (α’s) are used as the shape sensitivity measures, as inFreud and Behrmann (2020).

We first extracted SM’s head model from his structural MRI scan: the discretized brain model was extracted from the MRI scans using FreeSurfer (Dale et al., 1999;Fischl & Dale, 2000;Fischl et al., 1999a,1999b,^2001^,^2002^,^2004^). The CSF, skull, and scalp were extracted using the MNE software (Gramfort et al., 2013). With this extracted head model and using open-source MATLAB toolbox (FieldTrip;Oostenveld et al., 2011), we estimated the forward matrixA, needed by SilenceMap to estimate the source contribution measures (see equations (17) for low-resolution, and (35) for high-resolution source grid inChamanzar et al. (2021)for more details). We used a low-resolution source grid withp=804sources, and a high-resolution source grid withp=1608sources to estimate the source contribution measures.Figure 4shows the main two steps in our shape sensitivity analysis using SilenceMap.

#### 2.4.3 Step 1. Localization of ROIs across three levels

To provide a comprehensive analysis of the reduction in shape sensitivity, we used increasingly smaller and more precise regions of interest (ROIs) across three levels (seeTable 1for details). In*Step 1*, we first localize visually-responsive sources in healthy controls, which results in large regions, that is, Level 1 ROIs, that delineate all of the ventral and all of the dorsal cortex in each hemisphere. Since our specific interest in this study is in examining the shape sensitivity profile, we subsequently defined a set of six more narrowly-defined ROIs (see*Level 2 ROIs: two ventral and two dorsal regions*and*Level 3 ROIs: the right ventral lesion and its left homologous region*). This ROI definition is done using restricted anatomical regions from the Destrieux atlas (Destrieux et al., 2010) and intersecting the anatomical with the functional regions found in Level 1. In*Step 2*, we estimate the shape sensitivity slopes (α) within each localized ROI (seeSection 2.4.4).

**Table 1.** Overview of the ROI localization and shape sensitivity estimation steps across three levels.

Step 1: Localization of ROIs				Step 2: Estimation of slopes
ROI levels and descriptions		Data used to localize ROIs	Head model used to localize ROIs	Data used to estimate shape sensitivity slopes ( α )
Level 1	Broadest dorsal and ventral	Intact vs. resting state using data from each of the controls	SM’s head model	Scrambling levels vs resting state from each of the controls and SM within each ROI level
Level 2	Shape selective dorsal and ventral	Level 1 ∩ SM’s anatomical Destrieux parcellation	SM’s head model	
Level 3	Narrowest regions	Level 2 ∩ SM’s lesion and homologous area in Talairach coordinates	SM’s head model	

*Level 1 ROIs: finding sources that are activated by visual input.*In this step, we localize all regions in the brain with increased activity in response to visual input, in comparison to the activity in the resting state. Although SilenceMap is originally designed to localize neural silences, that is, regions with reduced neural activity, here, regions with increased activity can be localized as well. These regions were derived from the univariate contrast between the resting-state scan and the intact object condition (and not the other levels of scrambling).

**Fig. 5.**
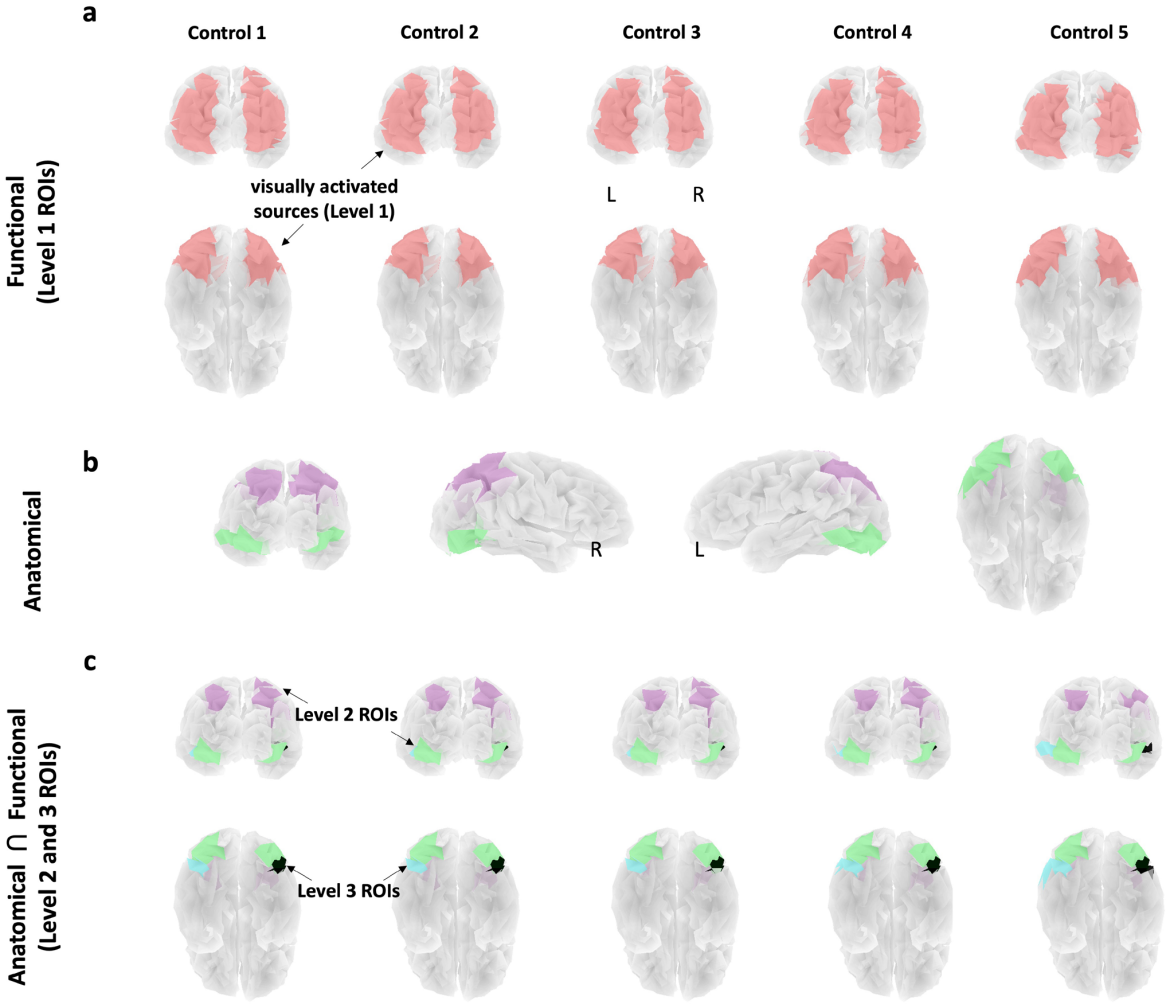
Regions of interest (ROIs) for shape sensitivity analysis. (a) Localized visually activated sources in five healthy controls. SilenceMap (Chamanzar et al., 2021) is used to localize contiguous regions with increased source contribution measured during the viewing of just the intact objects, in contrast to the resting state. The localized regions are mostly the same across healthy participants with small differences. This analysis is done based on full-band EEG ([1,50]Hz). These regions are the broadest dorsal and ventral ROIs for shape sensitivity analysis which we refer to as Level 1 ROIs (seeTable 1for details). (b) Anatomical parcellations of ventral (green region) and dorsal (purple region) ROIs in SM’s brain model. (c) Intersections of ventral and dorsal ROIs (b) with the visually activated sources in (a) for five controls. We refer to these more restricted dorsal and ventral regions as Level 2 ROIs. The right ventral lesion (black region) and its homologous region in the left hemisphere (blue region) are marked within the ventral ROIs, and are defined as Level 3 ROIs for shape sensitivity analysis.

Since the convex spectral clustering (CSpeC) framework in SilenceMap localizes one contiguous region with small$\beta_{q}$values (see equations (18) and (36) inChamanzar et al., 2021) as the region of silence, we (i) performed the localization task for each hemisphere separately, and assumed that there is only one contiguous region per hemisphere, and (ii) redefined the source contribution measure as follows:

$$\beta_{q}:=\frac{\beta_{q}^{rest}}{\beta_{q}^{S1}}=\frac{Var\left(\mu_{qt}^{rest}\right)-{\tilde{\text{a}}}_{q}^{T}{\text{C}}_{z}^{rest}{\tilde{\text{a}}}_{q}}{Var\left(\mu_{qt}^{S1}\right)-{\tilde{\text{a}}}_{q}^{T}{\text{C}}_{z}^{S1}{\tilde{\text{a}}}_{q}}\times \frac{{\tilde{\text{a}}}_{q}^{T}\left(\tilde{\text{A}}{\text{C}}_{s}^{full,S1}{\tilde{\text{A}}}^{T}\right){\tilde{\text{a}}}_{q}}{{\tilde{\text{a}}}_{q}^{T}\left(\tilde{\text{A}}{\text{C}}_{s}^{full,rest}{\tilde{\text{A}}}^{T}\right){\tilde{\text{a}}}_{q}},$$ (1)

where$\beta_{q}^{Rest}$is the source contribution measure for the resting state and$\beta_{q}^{S1}$is for the intact object condition. The source covariance matrix${\text{C}}_{s}^{full,rest}$is estimated based on the resting-state recordings using the least-square solutions in equation (34) inChamanzar et al. (2021), where$S_{elec}$includes the indices of all the electrodes on the scalp.${\text{C}}_{s}^{full,S1}$is estimated through iterations to find the visually activated region. The size of the localized region (k=100) is chosen to obtain a single contiguous solution from CSpeC for each hemisphere. This output resulted in masks large enough to cover both the dorsal and ventral pathways. We refer to these regions as Level 1 ROIs (seeTable 1for details).Figure 5ashows the localized visually activated regions in the five healthy controls. This step is performed on the full-band EEG ([1,50]Hz) to localize the Level 1 ROIs for the shape sensitivity analysis in*Step 2*, and SM’s extracted head model serves as the template head model for all participants. SilenceMap is robust to using template head models, as reported inChamanzar et al. (2021), and therefore this does not affect the reported results in this study. The results of shape sensitivity analysis in Level 1 ROIs are included inSection 3.1and summarized inFigure 7. As shown inFigure 5c, we further restrict these regions to obtain Level 2 and then Level 3 ROIs for shape sensitivity analysis.

*Level 2 ROIs: two ventral and two dorsal regions.*The extracted visually activated regions in Level 1 (Fig. 5a) are relatively large and may include both shape-sensitive and shape-insensitive voxels. To limit the scope of our analysis to the shape-sensitive voxels, we used anatomical information to arrive at a more tightly defined subset of dorsal and ventral ROIs (seeFig. 5b, c) and performed statistical analysis on shape sensitivity in SM. Needless to say, SilenceMap estimates the source contributions relative to a baseline, that is, Resting state (see*Step 2*for details). We specified ventral and dorsal regions in SM’s brain model, based on the anatomical Destrieux parcellation (Destrieux et al., 2010). Restricted anatomical regions 2 and 59 (i.e., inferior occipital gyrus and sulcus, and anterior occipital sulcus and preoccipital notch) were selected as the ventral regions, and 27 (superior parietal lobule) and 56 (intraparietal sulcus and transverse parietal sulci) were selected as the dorsal regions in each hemisphere (seefigure 2andtable 1inDestrieux et al., 2010for details). Cortical parcellation in SM’s brain model was extracted using FreeSurfer software (Dale et al., 1999;Fischl & Dale, 2000;Fischl et al., 1999a,1999b,^2001^,^2002^,^2004^).Figure 5bshows the extracted ventral and dorsal areas in SM’s brain model. For each control participant, we delineated the regions of overlap (i.e., Level 2 ROIs, seeFig. 5c) with the localized Level 1 ROIs. On average, the anatomical parcellation of ventral ROI (green region inFig. 5b) has 80% (∼39 voxels) overlap with the localized visually activated sources (red regions inFig. 5a), and the dorsal ROI (purple region inFig. 5b) has 35% overlap (∼27 voxels).

*Level 3 ROIs: the right ventral lesion and its left homologous region.*In Level 3 ROIs, we further limit the scope of our shape sensitivity analysis to the ventral lesion site and its homologous region in the RH. We used the Talairach coordinates (Talairach, 1988) of the lesion (+44, -46, -2) and its volume (990 mm^3^) fromKonen et al. (2011)to localize the lesion in SM’s brain model. We aligned the AC-PC line in the 3D brain model to derive the lesion’s Talairach coordinates. Source locations in the right ventral region (the Level 2 ROI) that are within a spherical volume of 990 mm^3^(4 voxels) around the lesion centroid were selected as the lesion ROI in our analysis (black ROIs inFig. 5c), and the mirror source locations in the left ventral region were used to define the LH homologous ROI (blue ROIs inFig. 5c). This spherical approximation of ROIs in neuroimaging studies is widely used and accepted (Poldrack, 2007;Strong et al., 2023). Our further investigation indicates that this spherical approximation of the lesion in SM does not affect the finding in this study. We have confirmed this by extracting the ground truth of the region of silence using AFNI and, critically, the results of the spherical approximation and ground truth are equivalent (seeSupplementary Note 1for details and results). The results of shape sensitivity analysis in Levels 2 and 3 ROIs are included inSection 3.2and summarized inFigure 8.

#### 2.4.4 Step 2. Estimation of shape sensitivity in localized ROIs

In this step, we estimated the shape sensitivity slopes (α) for each source within the localized regions extracted in the previous step. For each scrambling level, that is, S1 (intact), S4, S16, S64, and S256, we estimated the source contribution measures ($\beta_{q}^{Si}$) versus the resting state, that is,$\frac{\beta_{q}^{Si}}{\beta_{q}^{rest}}$. Therefore, this newly defined$\beta_{q,i}:=\frac{\beta_{q}^{Si}}{\beta_{q}^{rest}}$is the inverse of the definition inEquation (1), where${\text{C}}_{s}^{full,rest}$is estimated based on the resting-state recordings, as explained in*Step 2*, and${\text{C}}_{s}^{full,Si}$is estimated through the iterative steps in SilenceMap to localize the region of silence in each hemisphere. We expect that shape-sensitive sources show higher$\beta_{q,i}$values for lower scrambling levels and lower$\beta_{q,i}$ratio for higher scrambling levels, where the shape of the object is less recognizable.Figure 4bshows examples of this increasing trend of$\beta_{q,i}$fori=256,64,16,4,1in a healthy control for some of the sources in the visually activated region. The slope of$\beta_{q,i}$across the scrambling levels is estimated using a linear regression. The slope is then used for comparison of shape sensitivity of SM with controls at each source location ($\alpha_{q}$), and following the steps inFreud and Behrmann (2020)revealed reductions (Δα) in SM.

*Analysis of shape sensitivity reduction as a function of frequency bands.*We used the Crawford modified t-test (Crawford & Howell, 1998) to compare statistically the slope of shape sensitivity in each of the 5 frequency bands in each ROI between SM and controls. One-tailed modified t-tests were performed to determine whether there were significant differences in the slope between SM and the group of controls (with significance level of 0.05). To account for the multiple comparisons across the frequency bands, we also compared each of the controls to all other controls (leave-one-out) to determine whether the number of deviating frequency bands is greater/lesser in SM compared with the controls, creating an ad-hoc false discovery rate (Freud & Behrmann, 2020). Finally, using just SM’s data, we compared each frequency band pairwise against all others to evaluate whether any reduction in power as a function of shape sensitivity is selective to a particular frequency band. This was done using revised standardized difference test (RSDT,Crawford & Garthwaite, 2005). All the reported intervals in this paper are 95% confidence intervals, which are estimated using bootstrapping with a sample size of 1000. Employing Bayesian statistical approaches, such as Bayesian Crawford test (Crawford & Garthwaite, 2007), offers additional insights into the null-hypothesis inference and enhances the interpretability of the results. We performed Bayesian single-case testing as well as Bayesian Standardized Difference Test (BSDT) to estimate the percentage of the control population falling above or below the patient’s score. It is worth noting that the Bayesian Crawford single-case test does not provide a Bayes factor. However, to further bolster our observations in this study, we report whether SM’s data fall outside the 95% confidence interval of the group of controls. In Results, we only include the Bayesian test results for the significant (or marginally significant) observations to make the results concise. The full set of results for the Bayesian single-case and the BSDT test are included inSupplementary Data 2and3.

## 3 Results

We first confirmed that our paradigm replicated the linear modulation of activation as a function of the level of scrambling. In controls, this slope should be positive with greater amplitude as the display approaches the intact object condition (‘shape sensitivity’), except in the early visual cortex where this trend is reversed. The cortical maps from the controls inFigure 6, (α, measured in full-band EEG[1,50]Hz) replicate the fMRI results with clear shape sensitivity in both ventral and dorsal cortex in both hemispheres and also replicate those inCollins et al. (2019)using a similar paradigm.

**Fig. 6.**
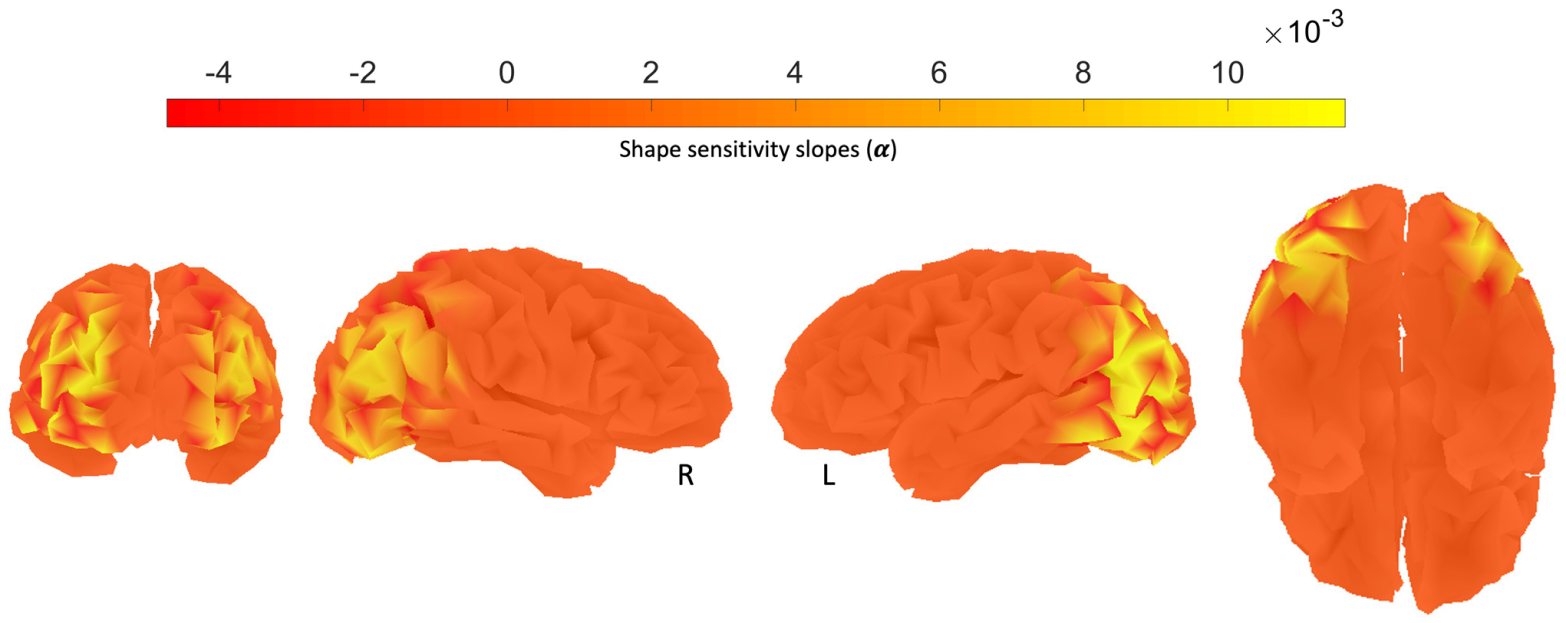
Cortical maps of average shape sensitivity slopes (α) for healthy controls in both ventral and dorsal cortex in both hemispheres. These shape sensitivity slopes are measured in full-band EEG ([1,50]Hz), contrasted against the resting-state data for each control separately. The resulting slope is then averaged across 5 healthy controls in this study.

### 3.1 Object shape sensitivity reduction through the lens of cortical maps in Level 1 ROIs

Having confirmed the robustness of the approach, we then compared the object shape sensitivity values for SM and each matched control, that is,$\Delta \alpha =\alpha_{\text{SM}}-\alpha_{\text{Ctr}{\text{l}}_{\text{i}}}$. The matrix in the left column ofFigure 7shows the cortical maps of shape sensitivity reduction in SM, in comparison to each of the five controls, separately for each of the five frequency bands. Negative values ofΔα($\alpha_{\text{SM}}<\alpha_{\text{Ctr}{\text{l}}_{\text{i}}}$, shown in warm red colors) indicate a reduction of object shape sensitivity in SM, while positive values ($\alpha_{\text{SM}}>\alpha_{\text{Ctr}{\text{l}}_{\text{i}}}$, shown in bright yellow colors) show an increase in shape sensitivity.Δαvalues are calculated in the Level 1 ROIs (i.e., visually responsive regions) extracted in*Step 1*of our analysis (seeFig. 5aandSection 2.4.3for details). The locations of reduction are consistent with the circumscribed structural lesion of SM (see the MRI scan inFig. 1) and with diaschisis in the homologous region of the structurally intact left hemisphere, as well as in dorsal regions. This diaschisis is discernible across most, if not all, frequency bands (quantified below). Some areas with increased shape sensitivity (yellow regions) are observed in SM in the right and left occipital and parietal regions, potentially reflecting compensatory activation of the early visual cortex perhaps as a result of repeated sampling of the high spatial frequency input as SM “problem solves” the input (Freud & Behrmann, 2020).

**Fig. 7.**
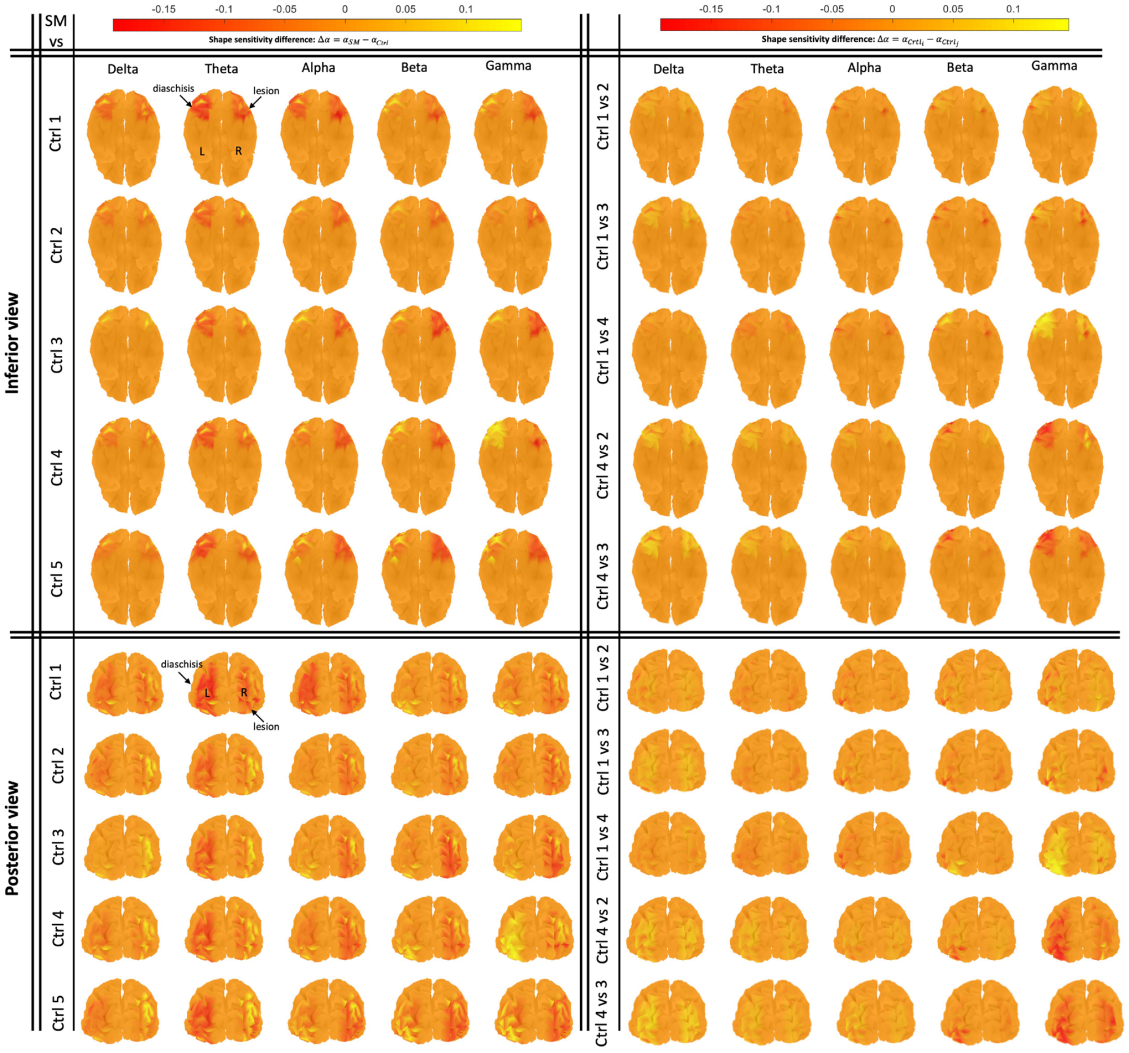
Cortical maps of shape sensitivity reduction (Δα) in Level 1 ROIs: Left column shows shape sensitivity reductions in SM, in comparison to five healthy matched controls (abbreviated as Ctrl), across different frequency bands of Delta ([1,4]Hz), Theta ([4,8]Hz), Alpha ([8,12]Hz), Beta ([12,30]Hz), and Gamma ([30,50]Hz).Δαvalues are calculated in the localized Level 1 ROIs (i.e., visually responsive regions) extracted in*Step 1*of our analysis (seeFig. 5aandSection 2.4.3for details). Negative values ofΔα($\alpha_{\text{SM}}<\alpha_{\text{Ctr}{\text{l}}_{\text{i}}}$, shown in warm red colors) indicate a reduction of object shape sensitivity in SM, while positive values show an increase in shape sensitivity ($\alpha_{\text{SM}}>\alpha_{\text{Ctr}{\text{l}}_{\text{i}}}$, shown in bright yellow colors). Shape sensitivity reductions in the posterior RH ventral region are observed across all frequency bands. This is consistent with the location of the structural lesion in SM based on his MRI scans (seeFig. 1). In addition, shape sensitivity reduction, which is more pronounced in the lower frequency bands (e.g., in Theta band), is observed in the homologous regions in the LH ventral and dorsal cortex in both hemispheres. Some regions with increased shape sensitivity (shown in bright yellow) are observed around the structural and functional lesions in both hemispheres which might be related to the compensation mechanism in SM for shape recognition impairments as explained inFreud and Behrmann (2020). The right column shows similar cortical maps of shape sensitivity reduction (Δα) for healthy controls, baselined against themselves ($\Delta \alpha =\alpha_{Ctrl_{i}}-\alpha_{Ctrl_{j}}$) as null distributions for the shape sensitivity analysis. Pairwise control comparisons are shown in inferior and posterior views across different frequency bands. No consistent pattern of shape sensitivity reduction is observed across the 10 comparison pairs and frequency bands as opposed to the pattern in SM which is replicable across comparisons with different controls.

The observed shape sensitivity reduction in SM is consistent and robust across comparisons with different controls (see the left column inFig. 7). To ensure that this shape sensitivity reduction pattern in SM is not a spurious effect of the comparison with the controls, we generated null distributions by repeating all the steps in our data analysis (see*Step 1–2*in Methods: Data analysis) for estimation of shape sensitivity difference (Δα), but now compared two controls at a time (i.e.,$\Delta \alpha =\alpha_{Ctrl_{i}}-\alpha_{Ctrl_{j}}$). This provides us with a set of cortical maps serving as a null distribution for shape sensitivity reductions, as shown in the right column inFigure 7, where there are no clear reductions in the control comparisons and no replicable effects in particular frequencies, as opposed to the consistent and replicable pattern of shape sensitivity reduction in SM (see the left column inFig. 7).

### 3.2 Statistical analysis of shape sensitivity reduction across different frequency bands and ROIs

**Fig. 8.**
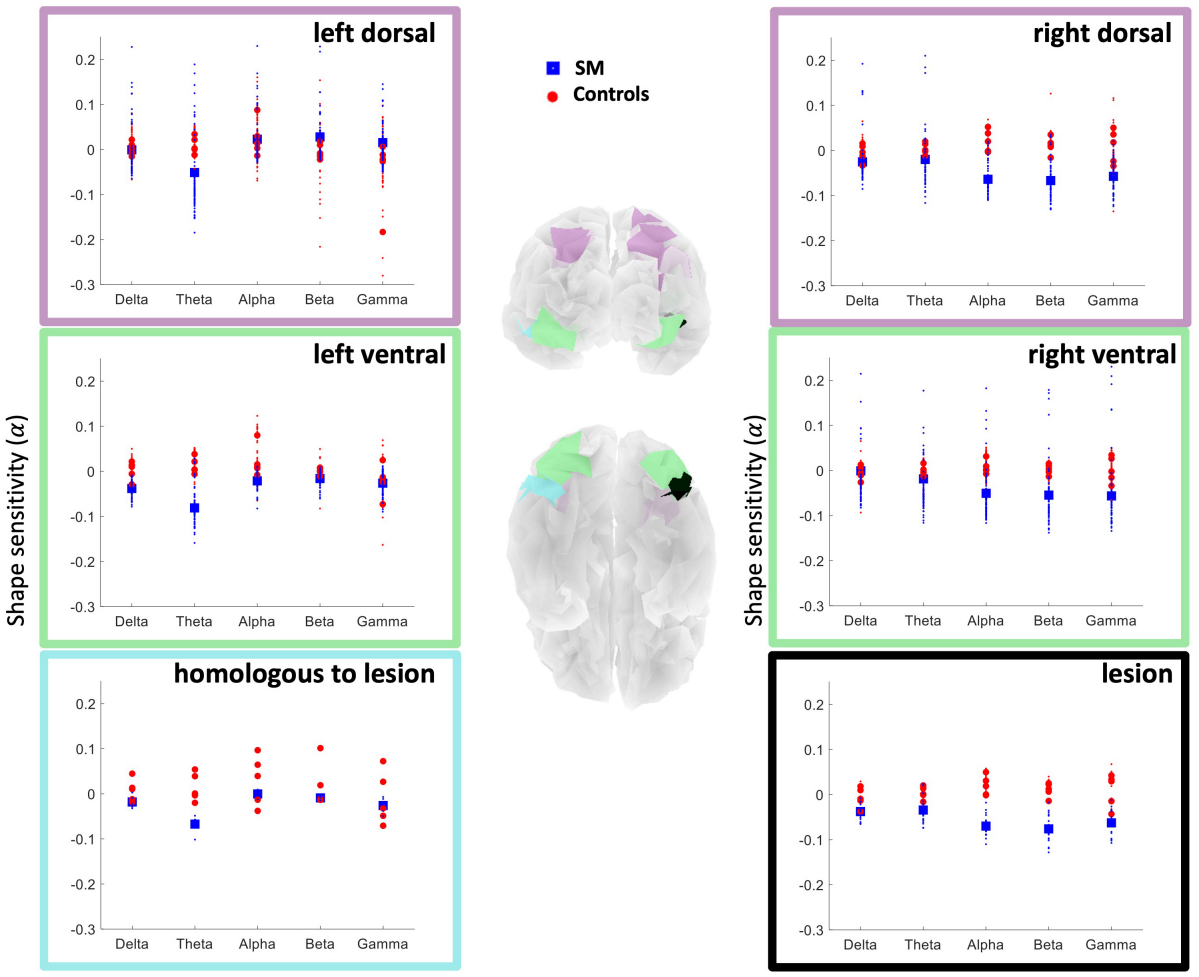
Comparison of shape sensitivity in SM versus controls across different frequency bands in LH (left column) and lesioned RH (right column): average shape sensitivity values (α) are shown within six ROIs, including Level 2 ROIs: left and right dorsal (panels with purple border) and ventral (panels with green border), and Level 3 ROIs: the right posterior ventral lesion in SM (the panel with black border) and its homologous ROI in the intact hemisphere (the panel with blue border). Mean shape sensitivity values at each ROI (averaged across the sources) are shown with blue squares for SM and red circles for the five controls at each of the five frequency bands of Delta ([1,4]Hz), Theta ([4,8]Hz), Alpha ([8,12]Hz), Beta ([12,30]Hz), and Gamma ([30,50]Hz). The shape sensitivity values of sources at each ROI are shown with small transparent dots in the background (red dots for controls and blue dots for SM). A shape sensitivity reduction in SM, in comparison to the healthy controls, is observed across all frequency bands in the right posterior ventral lesion (p<.037for Theta to Gamma andp<.066for Delta). The LH homologous region, namely, the remote effect of the lesion (diaschisis) in the intact hemisphere, shows a shape sensitivity reduction only in Theta band (p<.019). SM shows mostly negative shape sensitivity values in both dorsal and ventral regions (p<.04), except for Delta and Theta bands in the right dorsal, and Delta and Gamma bands in the left ventral cortex.

We then determined whether the reduction of the shape sensitivity in SM was statistically different from that of the controls. Because ventral and dorsal pathways are both involved in visual shape recognition, they are the most likely candidates for diaschisis (Freud & Behrmann, 2020;Freud et al., 2017b). Therefore, we performed one-tailed modified t-tests (Crawford & Howell, 1998) on the shape sensitivity values in SM in comparison to the group of controls in the anatomically restricted ROI levels (seeFig. 5candTable 1for the defined Level 2 and 3 ROIs in this study): We analyzed the data from Level 2 ROIs, that is, the intersection of Level 1 ROIs with dorsal (anatomical regions 27 and 56) and ventral (anatomical regions 2 and 59) ROIs in both hemispheres. Last, we examined shape sensitivity in SM versus the controls within Level 3 ROIs, that is, the intersection of Level 2 ROIs with the region of the right ventral lesion and its left homologous region.Figure 8shows these comparisons and the results are summarized below.

*Shape sensitivity reduction in Level 2 ROIs:*In scrutinizing the dorsal and ventral ROIs, a statistically significant reduction in shape sensitivity in the lesioned RH for SM versus controls was evident in Theta band (marginally significant in RH dorsal:$t_{\left(4\right)}=-1.51$,p=.051; significant in RH ventral:$t_{\left(4\right)}=-1.71$,p=.041), Alpha (RH dorsal:$t_{\left(4\right)}=-3.57$,p=.006; RH ventral:$t_{\left(4\right)}=-3.19$,p=.008), Beta (RH dorsal:$t_{\left(4\right)}=-4.61$,p=.002; RH ventral:$t_{\left(4\right)}=-3.89$,p=.004), and Gamma band (RH dorsal:$t_{\left(4\right)}=-1.86$,p=.034; RH ventral:$t_{\left(4\right)}=-1.64$,p=.044). A Bayesian test on the shape sensitivity reduction in Theta band in RH dorsal indicates that 10.1% ([0.1,40.2]%, 95% interval) of the control population falls below SM’s score. Bayesian Crawford does not confirm the null hypothesis for Theta in RH dorsal and Delta in area of lesion.

There was also a reduction in shape sensitivity in the structurally intact left ventral and dorsal ROIs, but this was only statistically significant in Theta (LH dorsal:$t_{\left(4\right)}=-4.84$,p=.002; LH ventral:$t_{\left(4\right)}=-3.01$,p=.01) and Beta band (LH dorsal:$t_{\left(4\right)}=-1.99$,p=.029; LH ventral:$t_{\left(4\right)}=1.72$,p=.04). This is consistent with the Theta band function reduction in the LH homologous region to the lesion (see below). In all of the significant and marginally significant reported results at the level 2 ROIs, SM’s score falls below and outside the 95% interval of controls. In both LH and RH dorsal ROIs, SM’s score falls outside the 95% interval of controls across multiple frequency bands, including Theta, Alpha, and Beta.

*Shape sensitivity reduction in Level 3 ROIs:*There was a significant reduction in shape sensitivity in SM compared to controls in all frequency bands, albeit marginal in the Delta band, at the lesion site (Delta:$t_{\left(4\right)}=-1.30$,p=.065; Theta:$t_{\left(4\right)}=-2.46$,p=.017; Alpha:$t_{\left(4\right)}=-3.81$,p=.005; Beta:$t_{\left(4\right)}=-5.03$,p=.002; Gamma:$t_{\left(4\right)}=-1.79$,p=.037). Bayesian Crawford test conducted on the shape sensitivity reduction in Delta band at the lesion site indicates that only 12.8% ([0.4,43.2]%, 95% interval) of the control sample falls below SM’s score. A significant difference between shape sensitivity of SM and the controls (SM<controls) was also present in the LH homologous region, albeit with a frequency-specific effect evident in Theta band ($t_{\left(4\right)}=-2.39$,p=.019). In all of the significant and marginally significant reported results at the lesion site and its LH homologous region, SM’s score falls below and outside the 95% interval of controls.

As evident inFigure 8, SM showed mostly negative shape sensitivity values (i.e., negative slopes across scrambling levels) in both dorsal and ventral regions (p<.04). The blue square in this Figure is SM’s slope of response signal across the different level of scrambling, calculated separately for each frequency band. The red symbols are the slopes for each of the controls. The increasingly negative slope values for SM are evident in all six ROIs at all frequency bands except for Delta and Theta bands in the right dorsal, and Delta and Gamma bands in the left ventral^1^. This indicates greater signal for more scrambled images, that is, more “edge sensitivity” than for more coherent images, that is, shape sensitivity, and is consistent with SM’s behavior and the self-report that he looks for features and then tries to build up the object from features.

Because we have compared one participant, SM, to the controls, it was important to conduct this case comparison with the controls as well. We performed a leave-one-out analysis by comparing each control against the rest of the controls (five comparisons) and then counted the number of frequency bands with a significant outcome (p<.05, one-tailed, Crawford t-test). Based on the results, there are 0.15±0.16 deviating frequencies in controls across the four ventral and dorsal ROIs. As reported above, SM showed 12 deviating frequencies, which is well outside the 95% confidence interval of the false discovery count.

*Is the shape sensitivity reduction in SM specific to particular frequency bands?*Here, we evaluated whether shape sensitivity in some frequency bands is more reduced than in others, relative to the same within-controls measures. We performed pairwise comparison of frequency bands in SM against the group of controls using the RSDT test with the two tasks being the two frequency bands (done for all pairs) for which we are comparing the difference in SM with controls. The shape sensitivity reduction in the right hemisphere did not differ as a function of frequency band but, in the LH, Theta band differs from Delta and Alpha frequency bands in the left Dorsal (Delta:$t_{\left(4\right)}=2.23$,p=.045; Alpha:$t_{\left(4\right)}=4.82$,p=.004) and Ventral regions (Delta:$t_{\left(4\right)}=2.36$,p=.039; Alpha:$t_{\left(4\right)}=2.87$,p=.023). Therefore, while the lesioned hemisphere shows a generalized shape sensitivity reduction regardless of the frequency band, the remote effect of the lesion is a frequency-specific Theta band reduction in shape sensitivity.

*Is the observed difference between SM and the controls specific to the shape sensitive ROIs in the brain?*We have documented significant differences in shape sensitive sources in SM in dorsal and in ventral regions. An obvious question is whether this finding is specific to these regions or is a property of all cortical regions, suggesting a generalized alteration in cortical dynamics. We, therefore, designed and performed an out-of-hypothesis ROI analysis for SM versus control differences in each of the five frequency bands: the first step was to define an out-of-hypothesis region that should not be affected by SM’s lesion and is both located out of the visual system and is not responsive to visual input. We chose dorsolateral prefrontal gyrus (DLPFC) using Destrieux anatomical regions 15 (middle frontal gyrus), 52 (inferior frontal sulcus), and 53 (middle frontal sulcus) similar to the definition of DLPFC inYamagishi et al. (2016). The resulting DLPFC ROI is shown inSupplementary Figure 2. Based on the Bayesian Crawford test results, there were no significant differences between SM and the healthy controls in the DLPFC ROI. This analysis reveals the specificity of the altered neural dynamics and diaschisis to the dorsal and ventral visual regions. This DLPFC analysis is included inSupplementary Note 2, and the Bayesian results are included inSupplementary Data 2.

## 4 Discussion

In this study, we conducted an analysis of the cortical electrophysiology of a single patient, SM, who, following a small lesion in the LOC of the right hemisphere (RH), presented with “visual agnosia”, a deficit in object and face recognition. Previously, in a task in which SM and matched controls viewed images of objects that were either intact or increasingly manipulated via a box scrambling algorithm, SM’s recognition performance was significantly poorer than that of controls, and this was so even when the displayed objects were entirely intact (Freud & Behrmann, 2020). In an fMRI study using the same parametric stimuli, SM’s BOLD activation profile (calculated as slope over increasing scrambling of images) in RH ventral/LOC lesion deviated significantly from the largely linear slope observed in controls. One standard interpretation of this agnosia deficit is to assume that it is the direct consequence of damage to a specific region such as his lesioned RH LOC. However, the atypical BOLD profile was also evident in the LH homologous ventral region as well as in the RH and LH dorsal cortices (Freud & Behrmann, 2020). These findings of remote effects (termed “diaschisis”;Finger et al., 2004) are consistent with the altered bilateral ventral signal reduction in SM, especially as complexity of the visual image increased, as shown in an earlier study (Konen et al., 2011). Other demonstrations of remote effects (Garcea et al., 2019) confirm a tightly coupled circuit between these pathways likely mediated by dense white matter tracts (Baizer et al., 1991;Yeatman et al., 2014) supporting the claim that even in cases of a circumscribed, focal deficit, the ripple effects can be observed in regions remote from the damage itself.

The focus of the current study is to elucidate the mechanism that might subserve the remote diaschisis. Using the same stimulus and design conditions as the box scrambling paradigm above, we collected high-density EEG data from SM. This allowed us to conduct a large-scale analysis of whole-brain signals as well as detailed temporal and spectral data that might explain the diaschisis. With the implementation of the SilenceMap algorithm (Chamanzar et al., 2021), we identified alterations in signals from ventral and dorsal sources both within the lesioned hemisphere and within the structurally preserved hemisphere. We also uncovered bilateral changes as a function of task demands (complexity of shape perception) and as a function of frequency bands. Together, the behavioral, EEG and fMRI findings are consistent with the claim that brain function derives from widespread interactivity across cortical regions and that behavior is governed by the joint activity of these networks (Vin et al., 2024;Wang et al., 2023;Zeraati et al., 2023). Taken together, the presence of these remote deficits is better accommodated by conceptual frameworks that assume distributed cortical circuits for behavior, including object recognition, rather than by an account that espouses a local region as the underlying neural substrate.

In the context of the distributed circuit, the remote effects of the lesion are conjectured to be mediated through the primary mechanism of functional deafferentation, that is, withdrawal of excitatory influences from anatomically connected regions of the brain (Boes & de Schotten, 2022;Siegel et al., 2022). Hypothetically, these remote effects may reflect “dynamic diaschisis” (Price et al., 2001), that is, they may be subject to task-dependent modulation. As revealed previously, the task-dependent diaschisis in SM is more pronounced when the difficulty of the visual task is increased (Konen et al., 2011). There are other reported findings which document task-specific alterations; for example, cross-hemispheric fMRI co-fluctuations in typical observers are noted in V1 when more taxing global stimulus configurations need to be assembled (Nasr et al., 2021).

### 4.1 Dynamic diaschisis in visual agnosia

EEG, with higher temporal resolution than fMRI or PET, has allowed us to uncover temporal and spectral dynamics of diaschisis and to elucidate the mechanism of the remote functional loss. In a series of analyses with different baselines and in comparison with control participants, we delineated several regions in which the electrophysiological response differed between SM and the matched controls. The key dependent measure we adopted was the slope of the power increase/decrease across levels of scrambling of the input from intact all the way to maximally scrambled (Freud & Behrmann, 2020;Freud et al., 2017b) computed separately in five frequency bands. A reduction in this shape sensitivity index in SM versus the controls was evident based on the signals extracted from the sources in the site of the lesion in the RH. This finding is entirely consistent with the ground-truth results previously obtained using fMRI data fromKonen et al (2011)andFreud and Behrmann (2020). In a more detailed analysis per frequency band from the lesion site, as well as the anatomically-defined ROIs of RH ventral and RH dorsal cortex (seeFig. 5c), the shape sensitivity reduction was present in Theta, Alpha, Beta, and Gamma bands (>4Hz). In contrast, the region homologous to the specific lesion site in the preserved LH ventral and dorsal regions, as well as in anatomically restricted ROIs of LH ventral and dorsal cortex (seeFig. 5c), all showed a reduction in shape sensitivity but only in Theta band (4−8Hz).

The flattening of the slope in the homologous LH region is consistent with previous neuroimaging data from SM (Freud & Behrmann, 2020;Konen et al., 2011) but also compatible with many demonstrations of transcallosal or interhemispheric diaschisis in other cognitive domains. In light of the abundant transcallosal connections between both hemispheres, opportunities for “reactive contralateral disinhibition” followed by contralateral hemispheric depression (Nguyen & Botez, 1998) are many. Bilateral reductions in function have been noted in motor (Volz et al., 2016) and language (Young et al., 2023) areas, and even in children who had suffered a perinatal stroke (Bhroin et al., 2021).

Modulation of diaschisis in select frequency bands in SM’s case offers a further perspective on diaschisis. The reduction in shape sensitivity in the affected hemisphere in both dorsal and ventral cortex was observed in multiple frequency bands. This widespread spectral functional loss in the lesioned hemisphere is consistent with the characteristics of the structural lesion with substantially suppressed neural activity and lack of function. As has been established previously (Gong & Zuo, 2023), the coarse skeleton of a functional organization hierarchy is present across multiple frequency bands, and, when a node is damaged, there is a cascading and ripple effect across the hierarchy. This is also consistent with findings that oscillatory brain interactions in source space during the recognition of familiar objects engage widespread reciprocal information flow (Supp et al., 2007). The novelty of the present findings is not only that signals from the RH lesion site, penumbra, and intrahemispheric connections are present in multiple frequency bands (Theta, Alpha, Beta, and Gamma) but also that the interhemispheric reductions (LH ventral and dorsal ROIs) are specifically demonstrated in Theta band.

### 4.2 The role of Theta oscillations in the context of cognitive functions

With respect to the interhemispheric alterations, in Theta band, electrophysiological studies have shown the importance of Theta band neural activities in several different cognitive functions (Buzsáki, 2005), including an increase in Theta in bilateral parieto-occipital regions during a visual working memory task (Moran et al., 2010), in a task-related fashion during neurofeedback, and during a working memory task (Klimesch, 2006). Also, bilateral intracranial recording from the hippocampus has implicated interhemispheric Theta coherence in an object-location memory task (Park et al., 2022) and, relatedly, Theta oscillation or the “navigation rhythm”, primarily originating from hippocampus, enables physical mapping and memory formation in the brain (Buzsáki, 2005). Theta oscillations are also associated with the ventral visual pathway both in the vigilance levels of visual spatial attention (Snipes et al., 2022), and in the context of face perception in the right occipital lobe (Dravida et al., 2019). Perhaps of most relevance here, Theta oscillation is hypothesized to be responsible for synchronization of neural firing across the cortex (Lisman & Jensen, 2013). As such, this interhemispheric role of Theta oscillation in cognitive and behavioral tasks, although understudied, may have the potential for the detection and characterization of transcallosal diaschisis.

A physiological explanation for these long range effects recognizes different processes of temporal dynamics, present in functional MRI BOLD signal but even more clearly manifest in electrophysiological measures with high temporal resolution (Mateo et al., 2017). The slow∼0.1 Hz resting-state functional connectivity noted in BOLD is attributed to vasomotion in the intrinsic oscillatory activities that link between functional and anatomical connections whereas the faster 30 to 80 Hz Gamma electrical activity is correlated with an increase with the BOLD signal per se (or decrease in the case of SM). Perhaps most relevant here are the temporal dynamics evoked in response to external sensory stimuli in which cerebral blood flow changes reflect an initial phase of deoxygenated hemoglobin followed by an active phase of neurovascular regulation of blood volume in the capillary bed, which occurs roughly on the time scale of the diaschisis effects observed in EEG and reported here. These changes may be observed in cross-hemispheric signals as well. Clearly, further research is needed to pinpoint the mechanisms that constrain signal propagation across brain regions both within- and between-hemispheres.

### 4.3 Visual agnosia and remote effects

There has been rather little consideration of remote effects of the cortical lesion in visual agnosia. For example, two studies which report EEG findings in pediatric visual agnosia cases focused on disordered continuous spike–wave discharges in slow sleep and changes in this profile over the course of treatment (Eriksson et al., 2003;van Iterson et al., 2021). Another study examined two patients, SM (who participated in this study) and JW, a patient with early visual cortex damage (Haigh et al., 2018). In this latter study, EEG data were collected while the patients viewed pattern-reversing checkerboards of differing spatial frequency. JW showed impaired steady-state visual-evoked potential response relative to a control group as well as lower decoding accuracy for early visual responses (around 100 ms). SM, whose lesion is located more anteriorly in the extrastriate cortex, showed good decoding accuracy early in the waveform but lower decoding after 500 ms. Although these findings do reveal altered EEGs in patients with agnosia, they do not bear on questions related to neural circuitry and remote effects, as is the focus of the current study.

The findings from the current study extend our understanding of the neural mechanisms underlying visual agnosia by demonstrating signal propagation abnormalities in a patient with a localized, unilateral brain lesion. Previous research, including studies with patient SM, has established that even unilateral lesions can lead to visual agnosia (Konen et al., 2011), indicating the significant role of distributed cortical networks in visual recognition. Our study advances this understanding by providing novel evidence that specific frequency bands, particularly those associated with long-range connectivity, are disrupted in SM.

The utilization of the unique SilenceMap algorithm allowed us to identify remote cortical regions exhibiting altered activity, supporting the concept of dynamic diaschisis and highlighting the importance of considering diaschisis when understanding the full impact of brain lesions on cognitive functions and behavioral outcomes. The use of high-density EEG, combined with the SilenceMap algorithm, represents a significant conceptual advance. It enables us to undertake a detailed examination of frequency-specific and task-dependent changes in cortical activity and has provided a nuanced view of the brain’s response to focal damage. Although this study only evaluated a single patient, the approach is readily generalizable to evaluate diaschisis in other patients, too. Future studies will be able to benefit from this methodological advancement to uncover the neural basis of other neuropsychological disorders.

### 4.4 Conclusion

The findings of widespread cortical dysfunction in an individual with a circumscribed unilateral lesion offer confirmatory evidence for theories espousing the critical nature of cortical connectivity within and between hemispheres. As such, these data challenge claims of more modular, discrete regions of cortex which, on their own, can lead to neuropsychological deficits. Instead, considerations of behavior-brain correspondences ought to account for the widespread consequences of a focal lesion and the difficulty disentangling the contribution of a single region to the observed behavioral deficit. Ultimately, predicting the consequences of damage in any region of cortex or behavioral domain will entail superimposing a patient’s lesion into the whole-brain connectome derived from typical subjects, and tracing the spread of the deficit in the context of ensuing widespread functional and structural connections, as has been done in the case of predicting remote effects post stroke (Salvalaggio et al., 2020;Siegel et al., 2022).

From a practical perspective, these results affirm the robustness of the SilenceMap algorithm for the spatiotemporal localization of multiple functional lesions in the frequency domain and its potential utility in delineating regions of dysfunction remote from the site of the lesion itself. Finding and demarcating the diaschisis and its task-based and spectral profile, in particular, has potential translational value. The use of neuromodulation intervention, for example, with transcranial magnetic stimulation, to regularize focal and non-focal neurophysiological changes distant to the lesion is considered a viable target of therapeutic strategies (Carrera & Tononi, 2014).

### 4.5 Limitations and future directions

An inherent limitation of EEG is its limited spatial resolution. SilenceMap achieves a spatial resolution of∼1cmin localizing regions of silence inChamanzar et al. (2021). This spatial resolution may not be sufficient for detection and characterization of very small functional loss or abnormalities in diaschisis. Although successful in estimation of shape sensitivity reduction in SM relative to controls and measuring normal shape sensitivity in controls across ventral and dorsal regions (Fig. 6), it may be insufficiently sensitive with more anatomically restricted regions such as the small RH homologous region of lesion, where for some controls close-to-zero or even negative shape sensitivities were estimated in some frequency bands (seeFig. 8). Further improvements in SilenceMap to better estimate the size and shape of the visually responsive regions used for shape sensitivity analysis might mitigate this limitation (seeFig. 4a). This would also enable the tailoring of ROIs for each control rather than the adoption of a generic mask (anatomical parcellation) of ventral and dorsal regions used in this study (Fig. 8b). Another limitation of SilenceMap is the ability to localize only a single contiguous region of silence per each hemisphere as accomplished with a careful use of baseline as explained in the Methods section. Extension of this technique to localize multiple regions of silence per hemisphere is, of course, intriguing, but beyond the scope of this paper. In addition, using multi-modal noninvasive imaging techniques such as EEG along with functional near infrared spectroscopy (fNIRS) can potentially improve our spatial resolution of signal reconstruction (Cao et al., 2023) and provide even more mechanistic perspectives on diaschisis such as further potential neurovascular couplings of contributing pathways to the functional loss.

Another obvious and interesting direction to pursue is the relationship between the ‘dynamic diaschisis’, in particular frequency bands and under particular conditions (evoked vs. rest) and structural measures. For example, it would be valuable to assay the corpus callosum and, using diffusion imaging, quantify the interhemispheric fibers/streamlines in the vicinity of the lesion (closer to the splenium) versus fibers at other loci of the callosum (closer to the genu). Observing no difference in callosal metrics would further limit explanations of dynamic diaschisis to the functional, rather than structural, domain. At the same time, quantification of white matter properties, for example, fractional anisotropy and diffusivity measures, between dorsal and ventral cortex regions defined here both within and between hemispheres would allow further inferences about diaschisis and integration of structural and functional metrics (see example of combined tractography with sEEG signals in explaining seizure propagation inAzeem et al., 2024).

A further interesting aspect of diaschisis which has not been fully explored is the spatio-temporal dynamics of signal propagation from the site of injury or lesion to the diaschisis region. Remote connections of synchronized regions in the brain are traditionally thought to be time- or phase-aligned (Gray et al., 1989;Muller et al., 2018;Uhlhaas et al., 2009). However, increasing evidence shows that the timing of neural synchronization constantly changes across time and space. This “flexible phase offset” in neural oscillation can be modeled as a traveling wave (Muller et al., 2018). These traveling waves can propagate in a single cortical region (i.e., “mesoscopic” waves) or across multiple regions (i.e., “macroscopic” waves) in the brain (Muller et al., 2018). Some examples of these waves are V1 visual waves as Gamma oscillations (30−80Hz;Besserve et al., 2015;Gabriel & Eckhorn, 2003;Vinck et al., 2010), sleep spindles (11−15Hz;Muller et al., 2016), or Hippocampal waves such as Theta oscillations (6−12Hz;Lubenov & Siapas, 2009;Patel et al., 2012;Zhang & Jacobs, 2015). Therefore, detection and tracking of spatio-temporal dynamics of oscillating phase offsets within and between cortical regions can potentially offer critical insights into the underlying mechanisms and pathways of diaschisis.

Last, measuring longitudinal changes in SM using the combined EEG and SilenceMap measures has the potential to shed light on any plastic or reorganizational changes in the distributed object recognition circuit. A reduction in the abnormality of remote effects might be indicative of positive changes and more normal functioning and might even have prognostic implications, as well. We do recognize the critical need to confirm our findings in a larger group of patients with agnosia (albeit a rather rare condition) or other neuropsychological conditions following a circumscribed lesion to the cortex. Additionally, adopting a larger group of control participants would add further robustness to our findings and approach. There are, however, demonstrations of diaschisis in several other neurological conditions (e.g., disruption in the attention network (He et al., 2007;Carter et al., 2010) or even motor network (Golestani et al., 2013;Park et al., 2011;Wang et al., 2010)), and given that many behaviors rely on a distributed neural circuit, the prediction is that dynamic diaschisis would be observed in these and many other circumstances, as well.

## Data and Code Availability

The anonymized raw EEG dataset (for both controls and SM) and MRI scans (only for SM) used in this research can be shared upon request.

SilenceMap was developed in MATLAB, using standard toolboxes, and the CVX MATLAB package (Grant & Boyd, 2008,^2013^). All MATLAB code is made available online on GitHub (DOI: 10.5281/zenodo.3892185;Chamanzar et al., 2020) following our publication inChamanzar et al. (2021).

## Author Contributions

A.C., M.B., E.F., and P.G. initiated, designed, and executed the research. A.C. and M.B. acquired and interpreted the data. M.B. and P.G. supervised the data collection. A.C. and P.G. developed the algorithms. A.C. developed the software tools necessary for conducting the experiments and analyzing the data. A.C., M.B., E.F., and P.G. wrote the manuscript.

## Declaration of Competing Interest

The authors filed a patent on the technology (Grover et al., 2024), assigned to Carnegie Mellon University. P.G. and M.B. are co-founders of a medical device company that intends to license the resulting patent from Carnegie Mellon University, and A.C. holds equity at this company.

## Supplementary Material

Supplementary Material

Supplementary Data 1

Supplementary Data 2

Supplementary Data 3

## Footnotes

1 LH dorsal, Delta:$t_{\left(59\right)}=-17.03$,p<.001; LH dorsal, Theta:$t_{\left(59\right)}=-16.10$,p<.001; LH dorsal, Alpha:$t_{\left(59\right)}=-9.38$,p<.001; LH dorsal, Beta:$t_{\left(59\right)}=-8.33$,p<.001; LH dorsal, Gamma:$t_{\left(59\right)}=-8.16$,p<.001; RH dorsal, Alpha:$t_{\left(73\right)}=-6.53$,p<.001; RH dorsal, Beta:$t_{\left(73\right)}=-6.43$,p<.001; RH dorsal, Gamma:$t_{\left(73\right)}=-6.53$,p<.001; LH ventral, Theta:$t_{\left(79\right)}=-4.05$,p<.001; LH ventral, Alpha:$t_{\left(79\right)}=2.71$,p=.008; LH ventral, Beta:$t_{\left(79\right)}=2.75$,p=.007; RH ventral, Delta:$t_{\left(59\right)}=-3.85$,p<.001; RH ventral, Theta:$t_{\left(59\right)}=-2.12$,p=.038; RH ventral, Alpha:$t_{\left(59\right)}=-15.59$,p<.001; RH ventral, Beta:$t_{\left(59\right)}=-12.71$,p<.001; RH ventral, Gamma:$t_{\left(59\right)}=-12.80$,p<.001.
