# Supplementary material for: Lesion-network mapping in task-dependent frequencies uncovers remote consequences of focal damage
Source: Imaging Neurosci (Camb). 2025 Apr 30;3:imag_a_00557. doi: 10.1162/imag_a_00557 (PMC12319782; doi:10.1162/imag_a_00557)
Supplement: Supplementary Material [file imag_a_00557-supp.pdf]

Supplementary Materials:
Lesion-network mapping in task-dependent frequencies uncovers remote consequences of focal damage

Alireza Chamanzar[1,2†], **Erez Freud**[3], **Pulkit Grover**[1,2*] **and Marlene Behrmann**[2,4,5*]
[1]Electrical and Computer Engineering Department, Carnegie Mellon University, Pittsburgh, PA, USA.
[2]Neuroscience Institute, Carnegie Mellon University, Pittsburgh, PA, USA.
[3]Department of Psychology and the Centre for Vision Research, York University, Canada
[4]Department of Psychology, Carnegie Mellon University, Pittsburgh, PA, USA.
[5]Department of Ophthalmology, University of Pittsburgh School of Medicine, Pittsburgh, PA, USA.
[†]correspondence to: achamanz@andrew.cmu.edu.
[*] Senior co-authors

This file includes:

- **Supplementary Note 1-2**
- **Supplementary Fig. 1-2**
- **Supplementary References**

Supplementary Note 1. AFNI-based lesion ROI extraction

The spherical approximation of ROIs in neuroimaging studies is widely used and accepted (Strong et al., 2023; Poldrack, 2007). 3D lesion and tumor extraction based on structural MRI is itself an active topic of research and beyond the scope of this paper. Efforts have been made using advanced deep learning techniques to do tumor segmentation based on MRI (e.g., the work by (Gunasekara et al., 2021)). However, to address this comment and make sure the spherical approximation of the lesion structure/shape does not affect our reported results, we worked to extract the 3D lesion ground truth. We used AFNI, an open source software developed by National Institute of Mental Health (NIMH) and specifically designed scripts, provided to us by Dr Daniel Glen (Computer Engineer, NIH), to segment the structural MRI scans of the patient in our dataset (Joachims, 1998; Cox, 1996). This function is designed to improve the segmentation of the scans in patients with lesions and/or tumors in their brain. It takes into account which hemisphere is intact in the brain, and along with a brain atlas (MNI152_T1_2009c+tlrc was used in our analysis), it strips the skull and segments the MRI scan. After segmentation of the structural MRI scans of SM in our study using AFNI, we extracted the ground truth region of silence (i.e., the right occipito-temporal lesion) following the steps in Section IV F of our previous paper (Chamanzar et al., 2021). We repeated all the analyses in the main manuscript using this extracted lesion ROI (see Supplementary Fig. 2). The results remain the same as reported in the paper (see Supplementary Data 1), which confirms that the spherical approximation of the lesion structure/shape does not affect our reported results.

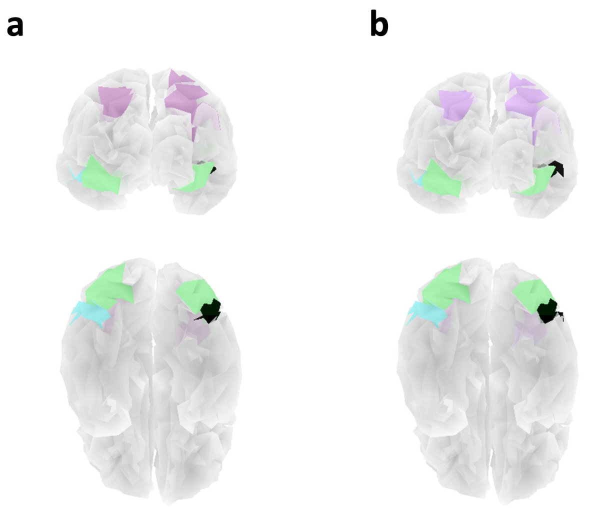

Supplementary Fig. 1. The right occipito-temporal lesion ROI in SM (black region): a) defined using a spherical ROI around the lesion centroid (Talairach coordinates +44, 46, 2 (Talairach, 1988)) and its volume (990 mm^3), and b) extracted based on AFNI MRI segmentation pipeline.

Supplementary Note 2. Out-of-hypothesis ROI analysis

One can think of our approach as specifically concerned with identifying effects that go beyond the lesion itself for the very reason raised in the paper (to be able to observe deficits remote from lesion site). We started by examining all brain regions which have a visual response (*Step 1.*) and limiting our analysis to these regions by intersecting with a structural mask. While it is hypothetically possible that regions outside of the visual cortex show differences in SM and controls, the goal was to examine the widespread circuit in dorsal and ventral visual responses. However, to show that the results we have obtained are specific to dorsal and ventral regions, we performed an out-of-hypothesis ROI analysis: The first step is to find a region for out-of-hypothesis that is expected to not be affected by SM's lesion at all. So it has to be located out of the visual system or visual responsive sources. Frontal lobe seems to be the best option. Since we are going to check both hemispheres, we would like a region that is equivalent across hemispheres. Many frontal regions may be affected by language, typically in the left hemisphere. Therefore, there are asymmetries in function already: if for example, we go with Broca's area in LH, its responses may be different from Broca in RH because it has its own specialization. To stay away from regions that might complicate this analysis and cause confusion, we choose DLPFC (dorsolateral prefrontal gyrus) using Destrieux anatomical regions 15 (middle frontal gyrus), 52 (inferior frontal sulcus), and 53 (middle frontal sulcus) similar to the definition of DLPFC in (Yamagishi et al., 2016). The resulting DLPFC ROI is shown in Supplementary Fig. 2. For this ROI, in the left and right hemisphere individually, we

compared the shape sensitivity slope in SM vs healthy controls in each of the five frequency bands. This is done, similar to the main analysis in the paper, using Bayesian Crawford t-test. Based on the Bayesian Crawford test results, there is no significant difference between SM and the controls in DLPFC.

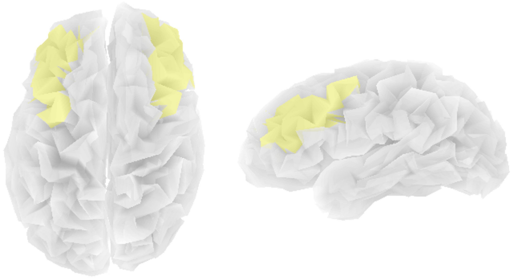

Supplementary Fig. 2. Out-of-hypothesis ROI in SM: the DLPFC (dorsolateral prefrontal gyrus, shown in yellow) was chosen and delineated using Destrieux anatomical regions 15 (middle frontal gyrus), 52 (inferior frontal sulcus), and 53 (middle frontal sulcus) similar to the definition of DLPFC in (Yamagishi et al., 2016).

Supplementary References

Chamanzar, A., Behrmann, M., and Grover, P. (2021). Neural silences can be localized rapidly using noninvasive scalp EEG. Communications biology, 4(1):1–17.

Cox, R. W. (1996). Afni: software for analysis and visualization of functional magnetic resonance neuroimages. Computers and Biomedical research, 29(3):162–173.

Gunasekara, S. R., Kaldera, H., and Dissanayake, M. B. (2021). A systematic approach for mri brain tumor localization and segmentation using deep learning and active contouring. Journal of Healthcare Engineering, 2021(1):6695108.

Joachims, T. (1998). Making large-scale svm learning practical. Technical report, Technical Report.

Poldrack, R. A. (2007). Region of interest analysis for fmri. Social cognitive and affective neuroscience, 2(1):67–70.

Strong, C. E., Zhang, J., Carrasco, M., Kundu, S., Boutin, M., Vishwasrao, H. D., Liu, J., Medina, A., Chen, Y.-C., Wilson, K., et al. (2023). Functional brain region-specific neural spheroids for modeling neurological diseases and therapeutics screening. Communications Biology, 6(1):1211.

Talairach, P. J. (1988). Co-planar stereotaxic atlas of the human brain. (No Title).

Yamagishi, T., Takagishi, H., Fermin, A. d. S. R., Kanai, R., Li, Y., and Matsumoto, Y. (2016). Cortical thickness of the dorsolateral prefrontal cortex predicts strategic choices in economic games. Proceedings of the National Academy of Sciences, 113(20):5582–5587.
